# Impact of sex on outcomes in septic shock patients treated with hydrocortisone

**DOI:** 10.1038/s41598-025-28014-5

**Published:** 2025-11-29

**Authors:** Hebatallah A. M. Moustafa, Mina Montasser, Islam Ahmed, Azza E. A. Mansy, Tamer Habib

**Affiliations:** 1 Clinical Pharmacy and Pharmacy Practice Department, Faculty of Pharmacy, Badr University in Cairo, Cairo, 11829 Egypt; 2 Emergency Medicine Department, Faculty of Medicine, Alexandria University, Alexandria, Egypt; 3 Pharmacy Practice and Clinical Pharmacy Department, Faculty of Pharmacy, King Salman International University, South-Sinai, Egypt; 4 Public Health and Community Medicine Department, Faculty of Medicine, Suez-Canal University, Ismailia, Egypt; 5 Clinical Pharmacy Department, Faculty of Pharmacy, Menofia University, Shibin al Kawm, Egypt; 6 Critical Care Medicine Department, Faculty of Medicine, Alexandria University, Alexandria, Egypt

**Keywords:** Septic shock, Corticosteroids, Hydrocortisone, Sex, Mortality, Prognosis, Medical research, Risk factors, Infectious diseases, Therapeutics

## Abstract

Limited evidence exists regarding the influence of sex on the prognosis of patients with persistent septic shock following hydrocortisone treatment. This study aimed to examine sex-related differences in hydrocortisone treatment outcomes among critically ill patients with persistent septic shock. This is a retrospective cohort study conducted over 12 months, including critically ill adult patients who were admitted with persistent septic shock and were on vasopressors while receiving hydrocortisone. The primary outcome was twenty-eight-day mortality. The secondary outcomes included vasopressor support duration, intensive care unit (ICU) stay, hospital stay, renal replacement therapy (RRT) requirement, mechanical ventilation (MV) requirement, and seven-day mortality. In total, 621 patients were analyzed; of these, 59.3% were male. Male sex was linked to an elevated 28-day mortality risk according to univariate analysis (OR 1.543, 95% CI 1.112–2.141; *p* = 0.009) and multivariate adjustment (OR 1.604, 95% CI 1.142–2.253; *p* = 0.006). According to the Kaplan–Meier curves, a significant difference in 28-day mortality (*p* = 0.008) was observed, with females exhibiting consistently higher cumulative survival rates. The initial analysis of secondary outcomes showed no differences, except for the requirement of renal replacement therapy (RRT); however, this association did not remain significant in multivariate analysis. This study underscores significant sex differences in the outcomes of patients with persistent septic shock treated with hydrocortisone, with males exhibiting higher 28-day mortality than females. In all measured secondary outcomes, no significant differences were detected.

*Trial registration*: This investigation was registered on clinicaltrials.gov (NCT06537180, study ID: GBD-HYDRO, 31 July 2024).

## Introduction

A complex network of immunological and circulatory dysfunction occurs in septic shock, increasing the likelihood of organ failure and death, and decreasing the quality of life for survivors^1–4^. Septic shock is treated with antibiotics, fluid resuscitation, vasoactive drugs, and mechanical organ support^5^. However, the dysregulated pro-inflammatory immune function of the host and its association with sex are not adequately addressed^6^.

Sex dimorphism may affect immunity, endothelial function, organ dysfunction, morbidity, and even mortality of septic shock^7–10^. Sex-related gene polymorphisms have been found to be associated with immune response in sepsis^11,12^. Differences in microbiome, late infection susceptibility, early clearance, and mortality were proposed to relate to the sex hormone effect in animal models^13,14^. Males have been reported to have a higher incidence of sepsis than females^15,16^. In addition, they were reported to be at higher risk for major infections, septic shock, multiple organ failure, and mortality^12,17–20^. Male sex hormones produce deleterious immunosuppressive actions and cardiovascular modulation in men after trauma or blood loss. On the other hand, female hormones are protective after trauma and hemorrhage^9^. Interestingly, the favorable effect of being a female on the prognosis of sepsis might be lost after menopause^21^.

Sex steroids directly and indirectly modulate the immune and cardiovascular responses. Sex hormones exert direct effects on the immune cells and cytokines such as interleukin-6, T lymphocytes, B lymphocytes, T helper cells, and natural killers via specific receptor-mediated processes. They also exert indirect effects during infections by modulating cardiovascular responses, immunity, and sex hormone-synthesizing enzymes^7,22,23^. The sensible administration of female hormones or the blockade of androgens was previously suggested for treating septic shock^24^. Several factors, such as stress, systemic inflammation, and body temperature, might cause circadian rhythm alterations in septic shock patients, affecting sepsis severity and the response to infection^25^. Interestingly, there is an association between body temperature, circadian rhythm, and sex influence on response to infection^26,27^. The evidence on the differences in thermoregulation between sexes due to anthropometric variations in adipose tissue and skeletal muscle mass may further explain the beneficial immune and physiological responses in females^28^, compared to males.

Because of their pleiotropic effects, corticosteroids are widely used as a concomitant treatment in sepsis for their anti-inflammatory, metabolic, and immune-suppressing actions^29,30^. Moreover, the treatment benefits of corticosteroids in terms of hemodynamics and organ functions for septic shock were established^31,32^. Hydrocortisone reduces mechanical ventilation (MV) duration, the duration of therapy within critical care, and the time needed for shock reversal^30^. When used in a daily dose of ≤ 400 mg for at least 3 days, it was associated with a survival benefit^33^.

Dehydroepiandrosterone (DHEAS) is a weak androgen that can be transformed by the action of aromatase to 17 β-estradiol and 5 α-reductase to dihydrotestosterone^34^. The better protection observed in women may be due to the greater peripheral fat tissue aromatase enzyme activity required for estrogen synthesis^34^. Several days of systemic inflammation often precede severe sepsis, which may decrease testosterone levels and increase estrogen production via aromatase activity^35,36^. Sex hormone levels might reflect an exhausted adrenal reserve in septic shock patients. Very low levels of the major circulating sex hormone in humans, dehydroepiandrosterone (DHEAS), were reported in septic shock patients, opposite to the increased cortisol levels. In addition, patients with relative adrenal insufficiency and those who didn’t survive sepsis had the lowest DHEAS values^37^.

The literature documenting the influence of sex on the prognosis of persistent septic shock after hydrocortisone use is sparse. The research sought to determine whether sex** i**mpacts the prognosis of hydrocortisone therapy in critically ill patients suffering from persistent septic shock**.** The hypothesis tested was that sex significantly affects the clinical outcomes following treatment using hydrocortisone in critically ill patients with persistent septic shock.

## Methods

Once authorization was granted by the local ethics committee of the Faculty of Medicine, Alexandria University, No: 0306756, and after obtaining a waiver for informed consent, the research was registered on clinicaltrials.gov (NCT06537180, study ID: GBD-HYDRO). We performed a single-center retrospective cohort study in the critical care units of Main Alexandria University Hospital, Egypt. The hospital has six main ICUs, from which data were collected from three: internal medicine, surgical, and urological. The internal medicine ICU has 40 beds, while the surgical and urological ICUs have 8 and 10 beds, respectively. Data of critically ill patients admitted with septic shock over 12 months (January 2023 – December 2023) was collected retrospectively. The following inclusion criteria were used: adult patients with persistent septic shock and requiring vasopressor therapy, defined as ≥ 0.25 mcg/kg/minute epinephrine or norepinephrine for ≥ 4 h after initiation of therapy^5^ who received hydrocortisone (200 mg/day IV, administered as a continuous infusion or 50 mg IV every 6 h)^5^ within the first 48 h from diagnosis. Patients with incomplete data sets were excluded.

Collected data included the following: demographic and epidemiological patients’ characteristics (age, sex, illness, medical history, and previous and current medications), routine laboratory investigations (complete blood picture, urea, serum creatinine, C-reactive protein, and procalcitonin), sepsis-related sequential organ failure (SOFA) score, acute physiology, and chronic health disease classification System (APACHE) II, the suspected source of sepsis, the vasopressor used, the duration of vasopressor, hospital stay, ICU stay, MV need, and RRT. All data were collected at a single point, “the initiation of hydrocortisone therapy”. The duration of using hydrocortisone and the associated adverse effects were recorded.

After data collection, patients were categorized according to their biological sex, which was recorded in their file, into 2 groups: males and females. The primary outcome of this study was 28-day mortality. The secondary outcomes included 7-day mortality, duration of vasopressor, ICU stay, hospital stay, need for MV, and the need for renal replacement therapy (RRT). The need for RRT was defined as “all patients who received RRT treatment (e.g., hemodialysis, continuous RRS) during their ICU stay according to local protocol and nephrology consultation”. We compared primary and secondary outcomes between groups. This work was reported per “Strengthening the Reporting of Observational studies in Epidemiology” (STROBE).

### Statistical analysis

The IBM SPSS (IBM Corp., Armonk, NY, USA) version 26.0 was utilized for analyzing data. We described qualitative variables as numbers and percentages. Quantitative variables were documented as ranges (minimum and maximum), means, standard deviations, and medians. We used the Kolmogorov–Smirnov test to check for normality. We described normally distributed continuous variables as the mean ± standard deviation (SD). We reported non-normally distributed continuous variables as the median ± interquartile range (IQR). We judged the significance of the results obtained at a 5% level. The chi-square test, Fisher’s exact test, Mann‒Whitney test, and Student’s t-test were used. The Hosmer–Lemeshow goodness-of-fit test was used for logistic regression. Log-rank test was utilized in the Kaplan–Meier survival analysis. The G*Power 3.1.9.7 was used for the post hoc analysis.

## Results

**Fig. 1:**
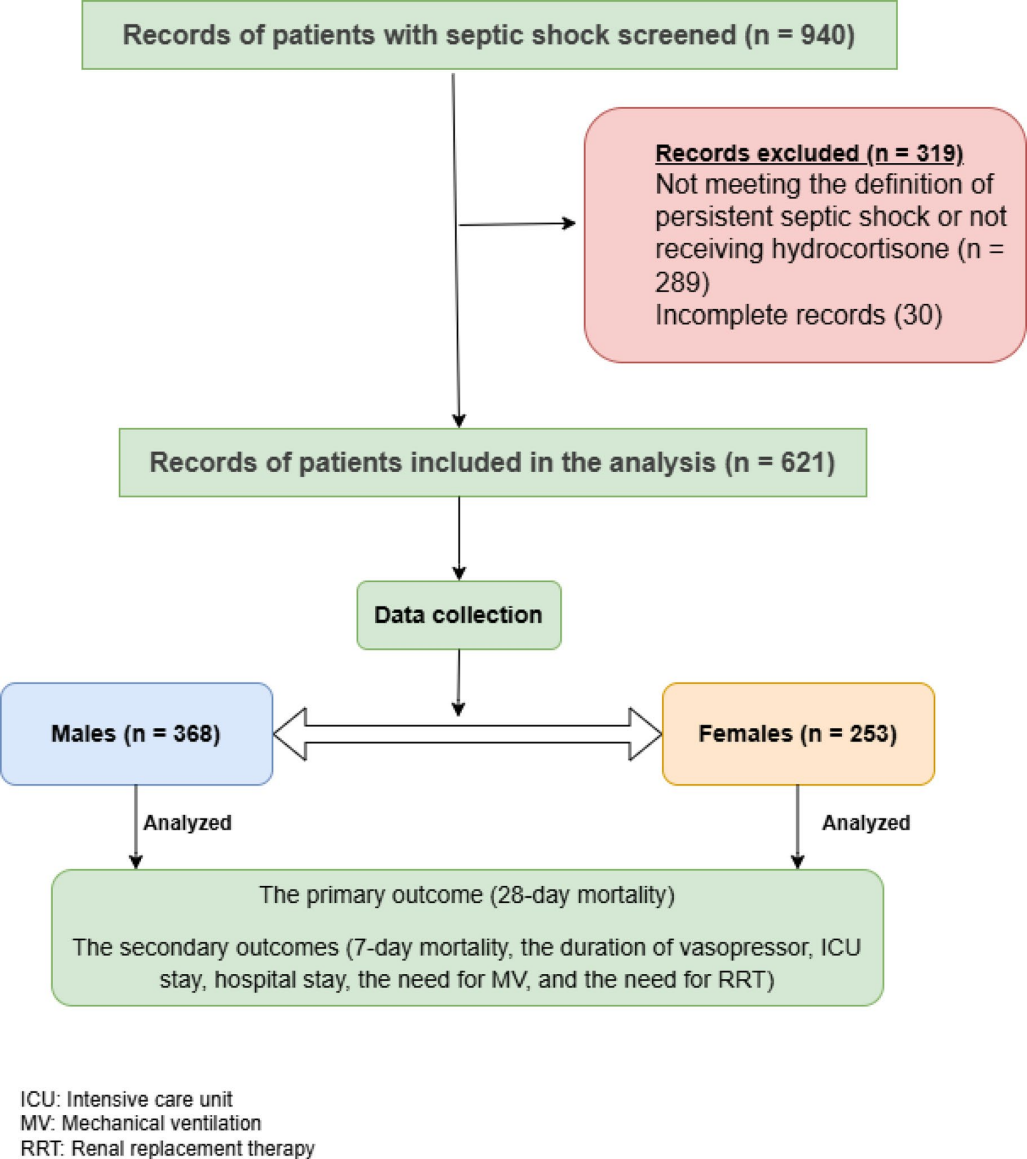
The STROBE flow chart of GBD-HYDRO trial.

In the current research, 940 septic shock patients were screened for analysis. After the exclusion of incomplete records and a lack of persistent septic shock definition, 621 patients were included. The majority of patients were enrolled from the internal medicine ICU (74.2%), followed by the surgical ICU (14.5%) and the urological ICU (11.23%). Males (59.3%) were more prevalent in this cohort. The mean age was 55.0 ± 6.2 years. No significant difference was found between females (54.8 ± 6.5 years) and males (55.1 ± 6.1 years, *p* = 0.563). The most prevalent medical history in the cohort was diabetes mellitus, affecting 50.7% of patients, followed by hypertension (46.2%) and COPD (32.2%). Notably, COPD was significantly more common among males (39.9%), compared to the female sex (21.0%, *p* < 0.005). Pneumonia was the most common source of sepsis (28.0%). Clinical severity scores, including SOFA (10.6 ± 2.1), APACHE II (21.2 ± 2.4), and Glasgow Coma Scale (11.5 ± 2.1), demonstrated no significant differences between both sexes (*p* > 0.05 for all). Routine laboratory investigations and sepsis markers were also comparable between the groups. No adverse events were reported after starting hydrocortisone (Table 1) (Fig. 1).

**Table 1:** The baseline characteristics of the studied patients.

	Overall (n = 621)	Female group (n = 253)	Male group (n = 368)	*p* value
Age (years)	55.0 ± 6.2	54.8 ± 6.5	55.1 ± 6.1	0.563
Medical history				
Hypertension	287 (46.2)	121 (47.8)	166 (45.1)	0.513
Diabetes mellitus	315 (50.7)	125 (49.4)	190 (49.3)	0.624
COPD	200 (32.2)	53 (21.0)	147 (38.1)	> 0.05*
Stroke	195 (31.4)	83 (32.8)	112 (29.1)	0.539
Hepatic	147 (23.7)	52 (20.6)	95 (24.7)	0.150
Atrial fibrillation	136 (21.9)	58 (23.0)	78 (20.3)	0.622
Malignancy	117 (18.8)	50 (19.8)	67 (17.4)	0.676
Ischemic heart disease	101 (16.3)	43 (17.0)	58 (15.1)	0.740
Recent Surgery	35 (5.6)	14 (5.5)	21 (5.5)	1.000
Source of sepsis				
Pneumonia	174 (28.0)	70 (27.7)	104 (27.0)	0.928
Urinary tract infection	152 (24.5)	63 (24.9)	89 (23.1)	0.850
Intrabdominal/SBP	117 (18.8)	56 (22.1)	61 (15.9)	0.095
Mixed source	50 (8.1)	20 (7.9)	30 (7.8)	1.000
Diabetic foot infection	53 (8.5)	19 (7.5)	34 (8.9)	0.469
Blood/CRBSI	43 (6.9)	14 (5.5)	29 (7.6)	0.334
Cellulitis	22 (3.5)	6 (2.4)	16 (4.2)	0.269
CNS infection	10 (1.6)	5 (2.0)	5 (1.3)	0.538
Glasgow coma scale	11.5 ± 2.1	11.4 ± 2.1	11.5 ± 2.1	0.404
SOFA score	10.6 ± 1.8	10.4 ± 1.8	10.7 ± 1.7	0.119
APACHE II score	21.2 ± 7.4	20.8 ± 7.5	21.5 ± 7.4	0.222
Hemoglobin (g/dl)	10.1 ± 2.7	10.0 ± 2.6	10.2 ± 2.7	0.516
WBCs (× 103 cells/ µl)	17.2 ± 8.7	17.5 ± 8.9	17.0 ± 8.6	0.571
PLTs (× 103/µl)	205.3 ± 83.8	206.8 ± 84.8	204.2 ± 83.3	0.706
INR	2.1 ± 0.7	2.1 ± 0.6	2.2 ± 0.7	0.171
Lactate (mmol/l)	5.2 ± 2.4	5.2 ± 2.5	5.3 ± 2.3	0.880
C-reactive protein (mg/l)	2.1 ± 0.7	2.1 ± 0.6	2.2 ± 0.7	0.171
Procalcitonin (ng/ml)	2.8 ± 1.6	2.7 ± 1.6	2.9 ± 1.6	0.185
BUN (mg/dl)	119.6 ± 72.1	117.4 ± 69.2	121.2 ± 74.1	0.514
Serum creatinine (mg/dl)	5.0 (6.3)	4.8 (5.5)	5.1 (6.8)	0.686
Sodium (mmol/l)	132.8 ± 9.5	132.8 ± 9.3	132.7 ± 9.7	0.930
Potassium (mmol/l)	4.0 ± 0.8	3.9 ± 0.8	4.1 ± 0.7	0.325

Categorical variables are expressed as number (percentage) and compared using the Chi-square test & Fisher’s exact test.

Normally distributed variables are expressed as Mean ± Standard deviation and compared using student’s t test.

Non-normally distributed variables are expressed as Median (Interquartile range) and compared using Mann–Whitney test.

AF, atrial fibrillation; COPD, chronic obstructive pulmonary disease; CKD, chronic kidney disease; IHD, ischemic heart disease; HTN, hypertension; DM, diabetes mellitus; SBP, spontaneous bacterial peritonitis; CRBSI, catheter-related blood stream infections; SOFA, sequential organ failure assessment score; APACHE II, acute physiology and chronic health evaluation version II score; WBCs, white blood cells count; PLTs, platelets count; INR, international normalized ratio; BUN, blood urea nitrogen.

*p value is significant when *p* < 0.05.

**Fig. 2:**
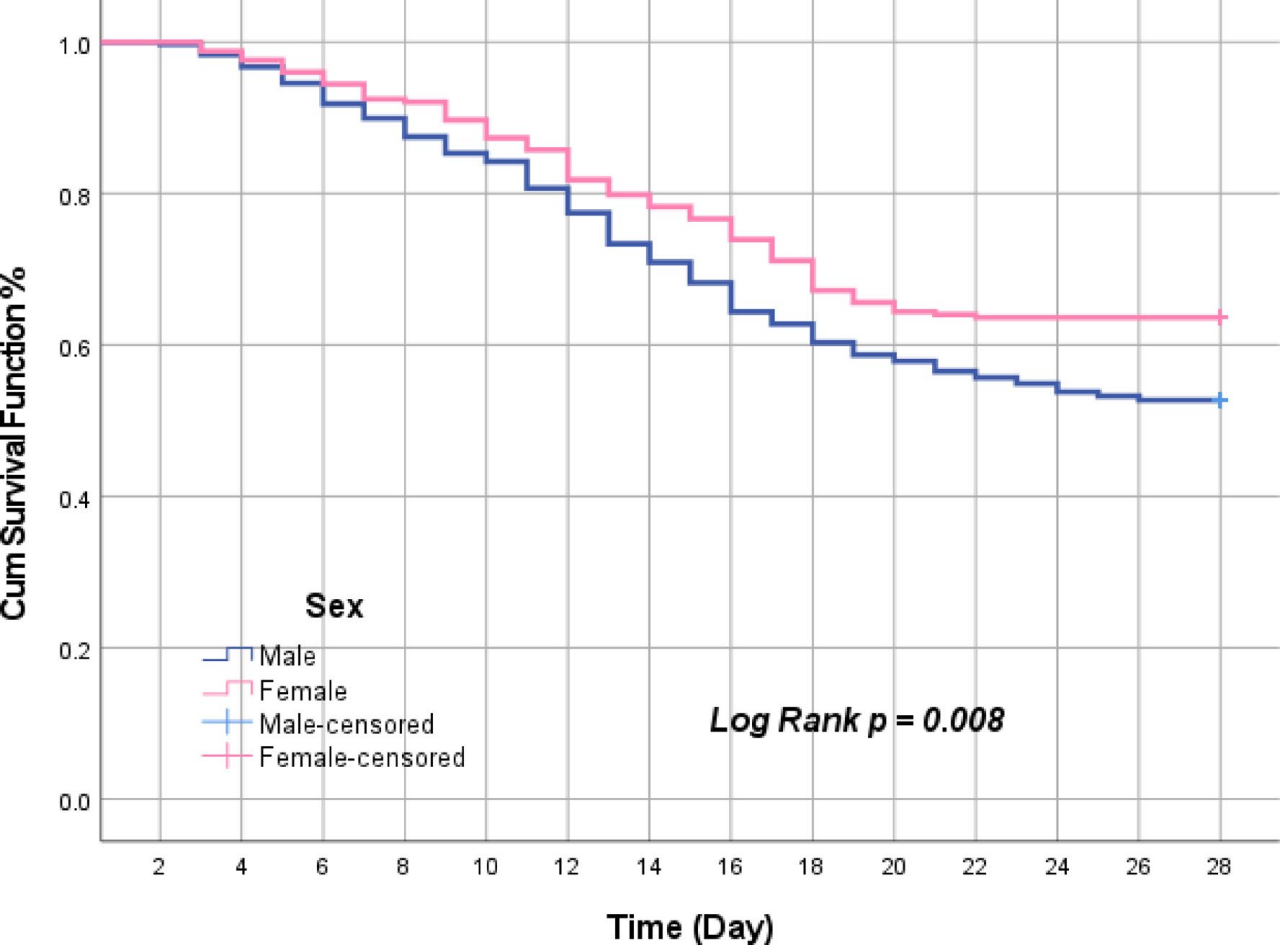
Kaplan–Meier survival curves for 28-day mortality stratified by sex.

The 28-day mortality, our primary outcome, was significantly increased in males (47.2%) versus females (36.7%, *p* = 0.011). In the univariate analysis, male sex was found to be significantly associated with increased 28-day mortality (Odds ratio = 1.543, 95% CI 1.112–2.141; *p* = 0.009) (Table 2). After adjusting for potential confounders and effect modifiers in the multivariable analysis, this association remained statistically significant (OR 1.604, 95% CI 1.142–2.253; *p* = 0.006) (Table 3). The Kaplan–Meier survival analysis showed a statistically significant difference in 28-day mortality between sexes (*p* = 0.008), in favor of females (Fig. 2).

**Table 2:** The measured primary and secondary outcomes.

	Overall (n = 621)	Female group (n = 253)	Male group (n = 368)	*p* value
28-day mortality	267 (42.9)	93 (36.7)	174 (47.2)	0.011*
MV days	9.2 ± 4.9	8.9 ± 4.8	9.4 ± 4.9	0.291
ICU days	13.8 ± 5.8	14.1 ± 5.9	13.5 ± 5.7	0.180
Need for RRT	356 (57.3)	133 (52.6)	223 (60.6)	0.048*
Vasopressor days	9.16 ± 4.9	8.8 ± 4.8	9.3 ± 4.9	0.232
7-day mortality	57 (9.2)	19 (7.5)	38 (10.3)	0.260

Normally distributed variables are expressed as Mean ± Standard deviation and compared using student’s t test.

Non-normally distributed variables are expressed as Median (Interquartile range) and compared using Mann–Whitney test.

MV, mechanical ventilation; ICU, intensive care unit; RRT, renal replacement therapy.

*p value is significant when p < 0.05.

**Table 3:** Multivariate-adjusted regression analysis for the primary outcomes.

Variable	Univariate analysis		Multivariate analysis	
	OR (95% CI)	*p* value	OR (95% CI)	*p* value
**28-day mortality**				
Male sex	1.543 (1.112–2.141)	0.009*	1.604 (1.142–2.253)	0.006*
SOFA score	0.947 (0.864–1.037)	0.238	0.936 (0.972–1.027)	0.162
APACHE II score	0.995 (0.974–1.017)	0.675	0.993 (0.972–1.015)	0.547
HTN	1.100 (0.800–1.513)	0.558	1.12 (0.808–1.553)	0.497
DM	1.158 (0.842–1.591)	0.367	1.176 (0.847–1.631)	0.333
Old stroke	1.036 (0.736–1.458)	0.840	1.047 (0.738–1.485)	0.798
CKD	1.043 (0.745–1.461)	0.806	1.018 (0.722–1.437)	0.917
COPD	1.031 (0.734–1.448)	0.861	0.959 (0.671–1.371)	0.818
Hepatic	1.107 (0.762–1.606)	0.594	1.078 (0.737–1.575)	0.70
Recent surgery	1.006 (0.505–2.004)	0.986	1.072 (0.522–2.199)	0.850
IHD	1.132 (0.737–1.737)	0.572	1.235 (0.795–1.918)	0.348
Chronic AF	1.018 (0.693–1.496)	0.926	0.995 (0.67–1.476)	0.979
Malignancy	1.482 (0.974–2.253)	0.066	0.663 (0.433–1.015)	0.059
Vasopressor days	0.978 (0.946–1.010)	0.175	0.974 (0.942–1.007)	0.123
**The need for RRT**				
Male sex	1.388 (1.004–1.917)	0.047*	1.386 (0.992–1.937)	0.056
SOFA score	1.016 (0.928–1.113)	0.734	1.015 (0.925–1.113)	0.754
APACHE II score	1.014 (0.993–1.036)	0.194	1.016 (0.994–1.039)	0.160
HTN	1.221 (0.887–1.679)	0.221	0.844 (0.609–1.168)	0.306
DM	1.184 (0.861–1.628)	0.298	1.194 (0.862–1.654)	0.286
Old stroke	1.122 (0.797–1.580)	0.508	0.966 (0.685–1.363)	0.435
CKD	1.027 (0.733–1.440)	0.876	0.870 (0.614–1.363)	0.844
COPD	1.020 (0.726–1.433)	0.910	0.87 (0.609–1.244)	0.579
Hepatic	1.190 (0.816–1.735)	0.367	1.19 (0.812–1.744)	0.373
Recent surgery	1.287 (0.650–2.548)	0.469	0.732 (0.36–1.489)	0.389
IHD	1.163 (0.753–1.797)	0.496	1.137 (0.729–1.773)	0.57
Chronic AF	1.082 (0.735–1.591)	0.690	1.053 (0.71–1.563)	0.796
Malignancy	1.218 (0.806–1.840)	0.349	1.219 (0.801–1.855)	0.354
Vasopressor days	1.002 (0.970–1.035)	0.894	1.002 (0.969–1.035)	0.916

Hosmer–Lemeshow goodness-of-fit model.

AF, atrial fibrillation; COPD, chronic obstructive pulmonary disease; SOFA, sequential organ failure assessment score; APACHE II, acute physiology and chronic health evaluation version II score; CKD, chronic kidney disease; IHD, ischemic heart disease; RRT, renal replacement therapy; HTN, hypertension; DM, diabetes mellitus; OR, Odds ratio; 95% CI, 95% confidence interval; LL, lower limit; UL, upper limit.

*Statistically significant at p ≤ 0.05.

A post hoc power analysis was performed for the logistic regression of 28-day mortality. The parameters used were: a two-tailed test; an OR of 1.604; a baseline probability of 28-day mortality for the reference group (females) of 0.367; an alpha error probability of 0.05; a total sample size of 621; an R^2^ from other covariates of 0.02 (derived from the Nagelkerke R^2^ of the regression model excluding sex); a binomial distribution for the predictor (sex); and a proportion of 0.593 for males in the sample. This analysis yielded an achieved power of 80.2%.

Regarding the secondary outcomes, the frequency of the need for RRT was significantly higher in males (60.6%) than in females (52.6%, *p* = 0.048). Other secondary outcomes, including the duration of MV, ICU stay length, vasopressor days, and 7-day mortality, demonstrated no significant variations between both sexes (*p* > 0.05 for all) (Table 2). The univariate analysis indicated an increased association of the need for RRT with male sex (OR 1.388, 95% CI 1.004–1.917; *p* = 0.047). Nevertheless, after controlling other variables in the multivariable analysis, no statistically significant association was detected. The lack of a standardized definition for RRT initiation in this retrospective study may have introduced variability in how RRT was assessed across samples, potentially contributing to the inconsistent findings.

## Discussion

To our knowledge, this study is the first sex-based investigation to examine the potential influence of sex on the prognosis of patients with persistent septic shock who are treated with hydrocortisone. The overall mortality rate in the current study was 42.9%, which is very close to previously reported rates in our institution^38,39^. Both univariate and multivariate analyses revealed that being male is independently associated with an increased risk of 28-day mortality. The Kaplan–Meier survival analysis corroborated this finding, showing a significant difference in 28-day mortality rates (*p* = 0.008). This observation suggests that females may derive greater benefits from initiating treatment with hydrocortisone compared to males. This difference may be attributed to the modulation of glucocorticoid receptor expression and sensitivity by sex hormones, which could potentially affect the body’s response to cortisol.

Although this study cannot fully determine whether the observed mortality difference was due to hydrocortisone, a recent review explored the mechanisms supporting better outcomes in females with septic shock, particularly in the context of corticosteroid treatment. Estrogen is known to exert protective effects against sepsis by modulating immune responses, acting primarily as an anti-inflammatory agent that dampens the expression of proinflammatory cytokines such as IL-6 and TNF-α, which are typically elevated during septic episodes. Females may exhibit more relative adrenal insufficiency. The presence of two X chromosomes in females allows for the expression of protective genes from the active X chromosome, potentially enhancing immune responses compared to males. Furthermore, the interplay between sex hormones and corticosteroids may influence treatment outcomes, as corticosteroids are known to modulate immune responses, and their effectiveness could be augmented in females due to estrogen’s influence on corticosteroid receptor activity. Moreover, while males often exhibit higher levels of proinflammatory cytokines, females may show elevated levels of anti-inflammatory IL-10. The protective effects of estrogen are particularly pronounced in premenopausal women, indicating that age and hormonal status are critical factors in determining outcomes in septic shock^40^.

Interestingly, Thompson et al.^41^ reported a sex difference in the cost-effectiveness of hydrocortisone treatment, suggesting that its cost-effectiveness may be more favorable for females with septic shock compared to males. This finding is attributed to higher mean utility values and lower total hospital costs associated with hydrocortisone use in females compared to placebo. The authors noted that the sex variation in the anti-inflammatory and immunologic responses to exogenous cortisol has not been previously evaluated.

A meta-analysis^42^ of eighteen randomized controlled trials showed conflicting results where corticosteroid therapy was related to a significantly shorter ICU stay among patients with septic shock. Results demonstrated that corticosteroids failed to decrease the 28-day mortality. In another meta-analysis^43^, results showed that the early administration of low doses of hydrocortisone can decrease short-term mortality risk in patients with septic shock. However, they did not perform sex-specific analysis. Multiple reports^44–49^ analyzed the impact of initiating early hydrocortisone in septic shock patients on reducing the vasopressor days, with conflicting results in terms of mortality. In these reports, females were underrepresented, as was observed in the findings. There was no sex subgrouping analysis in these reports.

In sepsis progression, sex differences in the hypothalamic–pituitary–adrenal (HPA) axis, cortisol metabolism, and immune responses are important parameters. Males are more susceptible to developing severe infections as bacteremia. In epidemiological studies, males showed higher rates of sepsis syndromes upon ICU admission^50,51^.

In the body’s stress response, the HPA axis is an important system and shows clear sex differences in baseline activity and stress. In females, this system is usually activated more rapidly and produces more hormones. While males often have stronger HPA responses to acute psychosocial stress. This includes higher cortisol and adrenocorticotropic hormone (ACTH) levels, with sharper increases. Gonadal hormones also contribute to these differences. Their effects start early at maturity and help shape the stress response system, which affects how humans respond to stressful conditions like sepsis. HPA function becomes more complicated in critical settings. During severe illness, cortisol levels cannot help controlling inflammation and do not meet the body’s metabolic needs, which can result in adrenal insufficiency. This variability is also affected by tissue-level corticosteroid resistance and biphasic HPA responses^52^.

In sepsis syndromes, the production and metabolism of cortisol are changed significantly. When compared to healthy individuals, patients show higher and more variable cortisol secretion rates and longer half-lives of free cortisol^53^. Although specific sex differences in these cortisol patterns during critical illness are not well described, existing knowledge about sex differences in HPA axis function and cortisol metabolism suggests that such variations are likely to exist. The ADRENAL trial showed that hydrocortisone increased shock recurrence risk in females, while it improved ICU discharge and weaning from ventilation in males. These inconsistent responses indicate that the effectiveness of corticosteroids in sepsis may depend on sex related metabolic pathways involved in glucocorticoid processing. If females convert more active cortisol to inactive forms, this could lessen the effectiveness of exogenous hydrocortisone. At this point, more research on sex differences in cortisol metabolism is encouraged^6^.

During infections, sex hormones significantly affect immune function, with clear differences between sexes. Androgens tend to suppress cell-mediated immunity. In contrast, estrogens improve humoral immune responses and overall immune defense, partly through the action of estrogen receptor β on immune cells. These effects are influenced by hormonal status and age. Additionally, sex-based differences in X-linked immune-related genes and mitochondrial metabolism create fundamental biological distinctions between both sexes that influence immune responses beyond the effects of hormones^50,54^.

This research has some limitations. The monocentric design and the lack of prior power analysis are limitations. The determination of sample size was not feasible due to the already reported difference in mortality between the two sexes in septic shock. A post hoc analysis was performed and revealed that the high-power estimates suggest that the study had sufficient statistical power to detect the reported association, reinforcing the robustness of the findings. However, while high power supports the validity of the significant results, it does not confirm the absence of effects for non-significant secondary outcomes such as MV days or ICU stay duration, which may have been influenced by lower power. The retrospective design lacks randomization and is liable to both selection and recall biases. The interindividual variabilities of initiating RRT were not controlled. Although the effects of the potential confounding variables were controlled in the multivariate logistic regression, other residual confounders may affect these study results. Finally, the incomplete records were excluded during the process of data collection, which can bias the results. Therefore, the generalizability of this study’s findings should be questionable.

## Conclusions

This study underscores significant sex differences in the outcomes of patients with persistent septic shock treated with hydrocortisone, with males exhibiting higher 28-day mortality than females. In all measured secondary outcomes, no significant differences were detected. These findings suggest that females may derive more mortality benefit from hydrocortisone treatment. These insights underscore the value of considering sex-specific responses during the management of septic shock and warrant further investigation into tailored therapeutic approaches.

## Data Availability

The data and material are available at reasonable requests from the corresponding author.
